# S204. NUTRITIONAL DEFICIENCIES AND CLINICAL CORRELATES IN FIRST-EPISODE PSYCHOSIS: A SYSTEMATIC REVIEW AND META-ANALYSIS

**DOI:** 10.1093/schbul/sby018.991

**Published:** 2018-04-01

**Authors:** Joseph Firth, Rebekah Carney, Scott Teasdale, Brendon Stubbs, Philip Ward, Michael Berk, Jerome Sarris

**Affiliations:** 1 University of Western Sydney; 2 University of Manchester; 3 UNSW and SESLHD; 4 Institute of Psychiatry, Psychology and Neuroscience King’s College London; 5 UNSW; 6 Deakin University, School of Medicine; 7 NICM, University of Western Sydney

## Abstract

**Background:**

Diet is increasingly recognised as a modifiable factor influencing the onset and outcomes of psychiatric disorders. Previous meta-analyses of blood nutrient levels in schizophrenia have already shown significant reductions in various individual vitamins/minerals. However, studies to date have largely focused on individual nutrients, and only considered nutrient status in patients with long-term schizophrenia. Meta-analytic evaluation of the evidence for nutrient deficits in first-episode psychosis (FEP) is completely absent. Therefore, we conducted a systematic review of all published studies comparing blood levels of vitamins and/or mineral in FEP to healthy control samples; and applied meta-analytic techniques to determine the prevalence and extent of deficiencies across the full spectrum of nutrients examined in this population to date.

**Methods:**

We searched electronic databases from inception to July 2017 for all studies examining blood levels (i.e. serum, plasma or whole blood) of nutrient levels in people with FEP compared to healthy controls. Our systematic search identified 28 eligible studies, examining blood levels of 16 different nutrients (six vitamins, ten dietary minerals) across 2,612 individuals: 1,221 patients with FEP and 1,391 control subjects. Random effects meta-analyses compared nutrient levels in FEP to healthy controls. Clinical correlates of nutritional status in patient samples were systematically reviewed.

**Results:**

Random effects meta-analyses found that people with FEP had large, significant reductions in blood levels of vitamin B9 (i.e. folate) compared to healthy controls(N=6, n=827, g=-0.624, 95% C.I.=-1.176 to -0.072, p=0.027). Significant reductions were also found for vitamin D (N=7, n=906, g=-1.055, 95% C.I.=-1.99 to -0.119, p=0.027) and, among fewer studies, vitamin C (N=2, n=96, g=-2.207, 95% C.I.=-3.71 to -0.71, p=0.004). No differences were found for other vitamins or minerals. Systematic synthesis of clinical correlates showed that reductions in both folate and vitamin D held significant relationships with greater psychiatric symptoms in FEP.

**Discussion:**

This is the first meta-analysis to examine the prevalence, extent and clinical correlates of nutritional deficiencies in FEP to date. The deficits in vitamin D and folate which have previously been observed in long-term schizophrenia appear to exist from illness onset, even prior to antipsychotic treatment, and are associated with more severe symptoms. The extent and importance of these deficiencies suggests that routine screening for vitamin D and folate deficiencies should be considered in early intervention services. Furthermore, since our previous meta-analyses have shown that high-dose b-vitamin supplementation can reduce symptoms in long-term schizophrenia, this should now be investigated in FEP. The potential physical and psychological benefits of vitamin D supplementation in early psychosis should also be explored. However, further research is needed to establish casual and mechanistic relationships between vitamin deficiencies, poor diet and the onset and outcomes of psychotic disorders.

